# Psychometric evaluation of the Participation and Activity Inventory for Children and Youth (PAI-CY) 0–2 years with visual impairment

**DOI:** 10.1007/s11136-019-02343-1

**Published:** 2019-10-31

**Authors:** Ellen B. M. Elsman, Ruth M. A. van Nispen, Gerardus H. M. B. van Rens

**Affiliations:** 1 Department of Ophthalmology, Amsterdam Public Health Research Institute, Amsterdam UMC, Vrije Universiteit Amsterdam, De Boelelaan 1117, Amsterdam, The Netherlands; 2 Department of Ophthalmology, Elkerliek Hospital, Wesselmanlaan 25, 5707 HA Helmond, The Netherlands; 3 Amsterdam UMC, VU University Medical Center, PK4X191, PO Box 7057, 1007 MB Amsterdam, The Netherlands

**Keywords:** Validation, Psychometrics, Children, Visual impairment, Item response theory, Participation

## Abstract

**Purpose:**

To identify and monitor the developmental and participation needs of visually impaired (VI) children, the Participation and Activity Inventory for Children and Youth (PAI-CY) has recently been developed involving end-users as stakeholders. The aim was to investigate psychometric properties of the PAI-CY for children between 0 and 2 years.

**Methods:**

Responses from 115 parents were included in item analyses, after which a combination of classical test theory and item response theory (IRT) was used. Internal consistency, known-group validity, and test–retest reliability at item and scale level were investigated.

**Results:**

After deleting four items, the PAI-CY met IRT assumptions, i.e., unidimensionality, local independence, and monotonicity, and satisfactory model fit was obtained. Participants with more severe VI and comorbidity scored significantly worse than those with less severe VI and without comorbidity, supporting known-group validity. Satisfactory internal consistency and test–retest reliability were obtained (Cronbach’s alpha 0.95, kappa 0.60–0.91, ICC 0.920).

**Conclusions:**

The PAI-CY 0–2 years has acceptable psychometric properties and can be used to systematically assess and monitor developmental and participation needs of very young children with VI from parents’ perspectives in low vision practice and research. Confirmation of psychometric properties is necessary, possibly facilitating further item reduction, increased precision, and improved user-friendliness.

## Introduction

Although the prevalence of childhood visual impairment (VI) is low [1], it has lifelong and far-reaching implications, for both children and their parents. According to parents of children with VI in the age band of 0–2 years and professionals with expertise in VI for this particular age group, sensory and general developmental issues related to attachment and well-being were among the most important concerns [2].

In the Netherlands, low vision services offer guidance such as developmental and behavioral interventions to overcome challenges related to vision loss. One of the most important outcomes of low vision services in children with visual impairment is participation, which for young children usually takes place in the family context [3]. In order to structure the process of identifying needs of children and their parents, the Participation and Activity Inventory for Children and Youth (PAI-CY) was recently developed involving end-users as stakeholders [2]. To aid interpretation, four different questionnaires were developed to reflect the developmental age bands of children as set by the World Health Organization (WHO). The PAI-CY should lead to a patient-based assessment of the impact of VI on functioning and participation and should initiate shared decision-making about interventions needed. Results from a pilot study showed that parents were mostly satisfied with the PAI-CY, whereas professionals suggested some changes which were incorporated in the next version [4]. The current study aims to investigate the psychometric properties of the PAI-CY 0–2 years.

## Methods

### Participants and procedure

Parents/caretakers (parents for brevity) of children aged 0–2 years registered at two Dutch low vision services were invited to participate. Parents who agreed to participate completed questions regarding socio-demographic and clinical information, the PAI-CY 0–2 years, and an evaluation form. Two weeks later [5], parents completed a retest. Although it should be noted that the very young age of children might result in less accurate data, ophthalmic diagnosis, visual acuity, and visual field of children were retrieved from patient files; missing values were complemented with self-reported data from parents (*n* = 10). VI was divided by five levels based on the better seeing eye and corresponding to the WHO criteria [6].

### PAI-CY 0–2 years

The preliminary version of the PAI-CY 0–2 years comprises 31 items categorized into seven domains (for descriptive purposes only, in order to provide contextual meaning): attachment, stimulus processing, visual attention, orientation, play, mobility, and communication that were informed by qualitative data from parents and concept-mapping workshops with professionals [2]. Each item is scored on a 4-point Likert scale with the following response options: not difficult (1), slightly difficult (2), very difficult (3), and impossible (4). The response option not applicable was treated as a missing value.

### Statistical analyses

Item analyses were conducted by examining missing responses and response category distributions. Items with missing scores > 20% were considered for elimination, as were items with > 70% of the respondents endorsing the first or last response category (i.e., floor or ceiling effects) and items having no answer in one of the response categories. Items showing inter-item correlations > 0.8, indicating potential redundancy, were also considered for elimination, as were items with an item-total correlation < 0.3. Cronbach’s alpha was calculated to evaluate internal consistency reliability.

An item response theory (IRT) model was subsequently applied. Items violating basic assumptions were considered for elimination [7–10]. Unidimensionality [11] was assessed by performing an eigenvalue decomposition on the matrix of robust (Spearman) correlations between the items. A difference approximation to the second-order derivatives along the eigenvalue curve (scree plot) was calculated. This acceleration approximation indicates points of abrupt change along the eigenvalue curve, and the number of eigenvalues before the point with the most abrupt change (the point with the maximum acceleration value) represents the number of latent dimensions that dominate the information content [7]. Subsequently, a principal component analyses (PCA) was performed to proxy if all items load on a single component (where the component is taken as a proxy for the latent trait). Magnitude of principal components was checked. Item pairs with excess covariation (> 0.25), signaling local dependence, were flagged. Monotonicity was assessed using Mokken scale analyses. The resulting graphs were visually inspected, and a Loevinger H coefficient was calculated to assess scalability (< 0.3 was considered unsatisfactory) [8–10]. The graded response model (GRM) was used [12, 13]; a full model was compared with a constrained model [11, 14, 15] nested within the full model with equal slope parameters across items. The models were compared using a likelihood ratio test (LRT). Relevant model fit indices were checked [16, 17]. Individual item performance was examined by assessing item fit using the *X*^2^ statistic. In addition, item and test information curves [11, 18, 19] were computed as well as an item-person map [20].

Known-group validity [5] was investigated using independent samples *t* tests and ANOVAs using post hoc Tukey tests for the following characteristics: gender, comorbidity, age (median split: ≤ 20 months vs. > 20 months), and level of VI according to the WHO criteria [6]. Test–retest reliability at item level was investigated using weighted kappa and percentage agreement [5, 21, 22] The intraclass correlation coefficient (ICC) for thetas calculated on test–retest data was based on absolute agreement in a two-way mixed-effects model.

All statistical analyses were conducted in R-Studio [23] and SPSS version 22 [24].

## Results

**Fig. 1:**
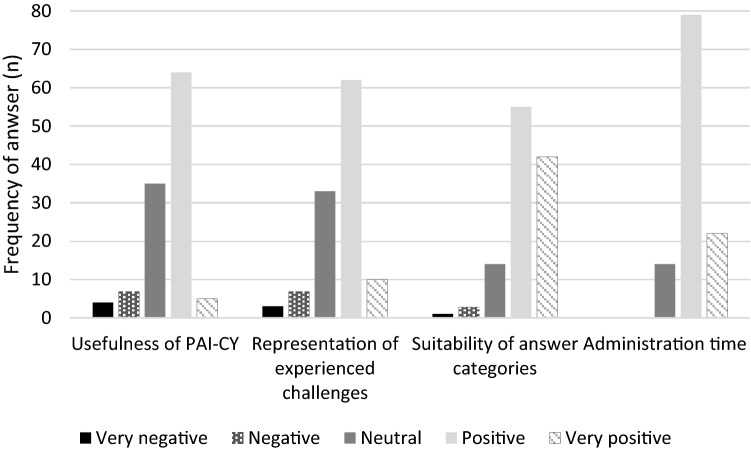
Evaluation of the PAI-CY 0–2 years by parents (*n* = 115)

Of an estimated 290 invited parents, 131 provided written informed consent to participate and completed the first questionnaire (45%). Data from participants > 25% missing responses on the PAI-CY 0–2 years (*n* = 14) or children > 2 years (*n* = 2) were excluded from the analyses. Table 1 presents characteristics of participants (*n* = 115). Out of the 115 participants, 54 had complete data on the PAI-CY 0–2 years, 45 respondents had one to three missing items, while nine respondents had > 5 missing items. Items were missing at random; there were no indications for acquiescence bias. Over 90% of the respondents were neutral to very positive about various aspects of the PAI-CY 0–2 years (Fig. 1), and no suggestions for improvements were made more than once. Self-reported administration time (including questions on demographic and clinical characteristics) was 17 ± 7 (range 5–40, median 15) min. The retest (*n* = 108) was completed after a mean of 30.5 ± 26.4 (range 11–181, median 19) days.

**Table 1:** Socio-demographic and clinical characteristics of participants (*n* = 115)

Age in months, mean ± SD (range)	20.91 ± 7.07 (7–35)
Male gender, *n* (%)	74 (64.3)
Severity of VI [5], *n* (%)	
No VI: logMAR ≤ 0.3	18 (15.7)
Mild VI: logMAR 0.31–0.52	14 (12.2)
Moderate VI: logMAR 0.53–1.00	46 (40.0)
Severe VI: logMAR 1.01–1.30	12 (10.4)
Blind: logMAR ≥ 1.31 or visual field ≤ 10°	15 (13.0)
Unknown	10 (8.7)
Comorbidity, *n* (%)	65 (56.5)
Parent who completed the questionnaire, *n* (%)	
Mother	90 (78.3)
Father	13 (11.3)
Mother and father together	11 (9.6)
Caretaker	1 (0.9)
Nationality parent, *n* (%)	
Dutch	110 (95.7)
Other	5 (4.3)
Education in years parent, mean ± SD (range)	13.54 ± 2.72 (0–16)
Financial situation parent, *n* (%)	
Usually enough money	65 (56.5)
Just enough money	26 (22.6)
Not enough money	5 (4.3)
No answer	19 (16.5)

The items “reacting to (sudden) sounds” and “entertaining alone” were deleted because of low factor loadings, low information, and low scalability coefficients. “Looking at a particular item nearby” and “following a toy or person with the eyes” were deleted because of local dependence with too many other items. Furthermore, omitting these items was considered not to violate content validity, because they were thought not to be suitable for the target population (entertaining alone) or because of similarity to other items.

None of the items had disturbing amounts of missing responses according to our cut-off criterion, whereas three items demonstrated floor effects. In general, the fourth answer category was infrequently endorsed, but collapsing answer category 3 and 4 led to questionable unidimensionality, as the relative increase of the explained variance of the second factor was limited compared to the first factor. Therefore, the response categories of only a few items were collapsed (indicated in bold in Table 2). Four item pairs showed high inter-item correlations (Table 2), whereas none of the items had item-total correlations < 0.3. Cronbach’s alpha was 0.95.

**Table 2:** Distribution of responses over the response categories, GRM item parameter estimates, item information, item fit, and parameters for test–retest reliability for the PAI-CY 0–2 years

Domain and item content	Missing (%)	Distribution of responses over response categories (%)*				GRM item parameter estimates				Item information over entire score range (− 10; 10)	Item fit		Test–retest reliability parameters	
		1	2	3	4	*α*	β1	β2	β3		*Χ*^2^	*P* value	Agreement %	Weighted kappa
1: Recognizing facial expressions^a^	9.57	45.19	25.96	20.19	8.65	1.46	− 0.44	0.59	1.91	2.97	12.52	0.09	67.4	0.76
1: Imitating facial expressions^a^	11.30	33.33	25.49	27.45	13.73	2.09	− 0.80	0.06	1.35	4.74	10.41	0.11	69.6	0.82
1: Recognizing faces of familiar people	5.22	43.12	33.03	**14.68**	**9.17**	0.91	− 0.59	1.28		1.39	4.84	0.94	60.6	0.67
1: Imitating actions or behavior	0.87	53.51	**17.54**	**18.42**	10.53	2.52	− 0.12	1.48		4.76	5.13	0.28	77.1	0.75
1: Imitating sounds	1.74	55.75	**16.81**	**18.58**	8.85	1.67	0.04	1.94		3.01	4.14	0.39	86.5	0.85
1: Exploring the environment	0.00	43.48	31.30	16.52	8.70	2.10	− 0.43	0.72	1.78	4.57	3.50	0.62	74.0	0.85
2: Reacting to visual stimuli	0.87	70.18	20.18	**5.26**	**4.39**	1.48	0.68	1.99		2.33	0.64	0.89	77.4	0.60
2: Recognizing familiar sounds	4.35	76.36	16.36	**4.55**	**2.73**	1.12	1.15	2.61		1.67	2.03	0.16	83.2	0.68
2: Reacting to sudden actions^b^	0.87	52.63	32.46	14.04	0.88	0.49	0.04	3.52	9.70	0.90	18.35	0.02	67.3	0.66
3: Looking at an item further away	11.30	6.86	23.53	28.43	41.18	0.98	− 3.21	− 1.24	0.23	1.98	18.37	0.03	64.4	0.79
3: Looking at something for longer time	2.61	21.43	37.50	35.71	5.36	0.92	− 1.80	0.35	3.44	2.14	12.00	0.29	58.7	0.69
3: Alternating visual attention	3.48	43.24	35.14	18.02	3.60	1.34	− 0.50	1.12	2.97	3.10	6.51	0.37	76.9	0.84
4: Orienting in a room^c^	8.70	33.33	43.81	18.10	4.76	1.15	− 0.98	1.08	2.97	2.64	14.93	0.06	66.0	0.76
4: Exploring the environment by touch	4.35	60.91	23.64	10.00	5.45	1.77	0.15	1.29	2.30	3.78	4.16	0.25	71.7	0.66
5: Looking at pictures together^d^	1.74	38.94	36.28	15.93	8.85	1.54	− 0.61	0.89	2.00	3.36	6.91	0.33	60.8	0.75
5: Reading books together^d^	6.96	37.38	41.12	14.95	6.54	1.46	− 0.70	1.01	2.24	3.26	9.80	0.13	57.3	0.70
5: Manipulating toys	0.87	42.98	33.33	18.42	5.26	2.04	− 0.43	0.84	2.17	4.96	4.59	0.33	68.2	0.69
5: Playing with children of similar age	14.78	29.59	31.63	22.45	16.33	2.06	− 0.88	0.20	1.15	4.58	4.22	0.52	70.1	0.84
6: Raising the head	5.22	75.23	14.68	8.26	1.83	2.57	0.71	1.55	2.69	6.11	1.11	N/A	89.0	0.86
6: Rolling over	4.35	69.09	16.36	12.73	1.82	2.33	0.48	1.25	2.76	5.54	1.68	0.20	84.2	0.76
6: Crawling, tidying, sliding buttocks^e^	6.09	57.41	**12.96**	**13.89**	15.74	3.32	− 0.03	1.10		6.18	6.22	0.05	86.5	0.88
6: Sitting	2.61	67.86	12.50	10.71	8.93	3.60	0.32	0.84	1.50	8.02	2.64	N/A	84.3	0.91
6: Pulling to stand^e^	6.09	50.93	**12.96**	**16.67**	19.44	3.40	− 0.24	0.84		6.32	6.46	0.09	82.7	0.84
6: Standing independently^f^	13.04	42.00	17.00	15.00	26.00	3.21	− 0.49	− 0.00	0.54	6.45	4.28	0.04	90.0	0.88
6: Walking with support^f^	14.78	45.92	**14.29**	**14.29**	25.51	2.51	− 0.34	0.64		4.22	9.05	0.11	75.3	0.82
7: Understanding simple gestures	4.35	51.82	20.00	17.27	10.91	2.23	− 0.19	0.53	1.49	4.73	7.69	0.17	73.0	0.81
7: Understanding language	4.35	57.27	22.73	12.73	7.27	2.58	− 0.02	0.85	1.72	5.91	3.90	0.27	78.0	0.88

1: Attachment; 2: stimulus processing, 3: visual attention, 4: orientation, 5: play, 6: mobility, 7: communication

*Values displayed in bold represent categories which were collapsed

^a^Items showed local dependence (0.317) and high inter-item correlation (0.833)

^b^Item showed unsatisfactory PCA loading (0.30) and scalability (0.29)

^c^Item violated the monotonicity assumption

^d^Items showed local dependence (0.309) and high inter-item correlation (0.846)

^e^Items showed high inter-item correlation (0.913)

^f^Items showed local dependence (0.325) and high inter-item correlation (0.903)

The assumptions for IRT seemed to hold for most items (Table 2 shows items violating assumptions). The items represented a unidimensional model; a one-factor model explained 48% of the variance and mostly yielded high component loadings (> 0.45). A two-factor solution added 10% explained variance. The ratio of 4.8 between the first and second factor is higher than the required minimum of 4 [25]. Out of 351 possible item pairs, three item pairs violated the local independence assumption. One item violated the monotonicity assumption and one item had an insufficient scalability coefficient. It was decided not to delete these items because of content relevance.

**Fig. 2:**
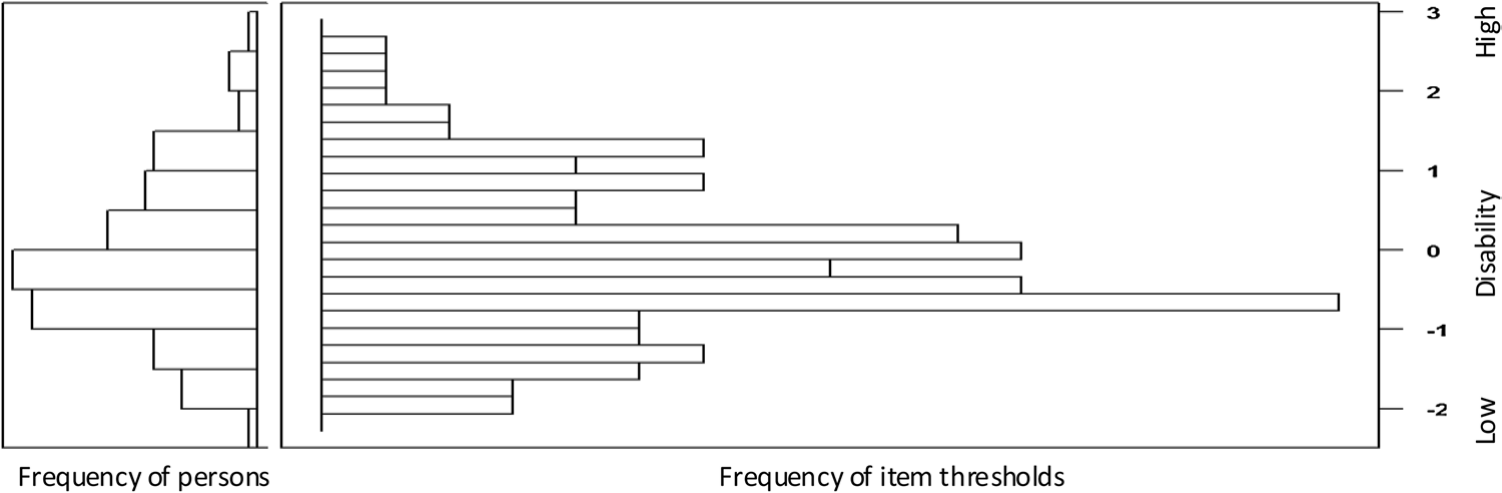
Item-person map of the PAI-CY 0–2 years

The full GRM outperformed the constrained model (LRT = 116.42, df = 26, *p* < 0.001), and model fit approached satisfactory values (RMSEA = 0.064, SRMR = 0.099, TLI = 0.965, CFI = 0.968). Item parameters, information, and fit statistics are displayed in Table 2. We confirmed the validity of the IRT parameters by examining the differences in test–retest parameters, which were on average small (*α*: 0.53 ± 0.48, *β*1: 0.21 ± 0.17, *β*2: 0.29 ± 0.27, *β*3: 0.43 ± 0.32); note that test and retest data are highly correlated (*r* = 0.92). Despite the fact that some items provided little information, all items were maintained, because removing the item with lowest information (“reacting to sudden actions”) resulted in more violations of assumptions for the remaining items. Difficulty of items matched respondents’ ability (Fig. 2).

**Fig. 3:**
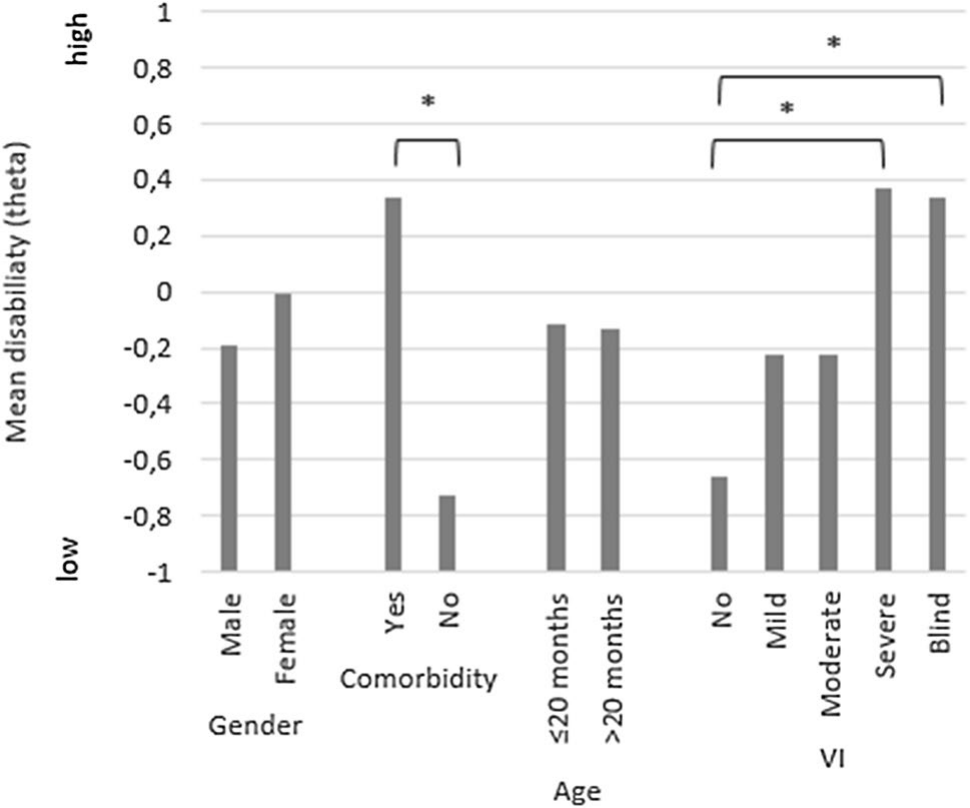
Mean disability (theta) by gender, presence of comorbidity, age, and severity of VI. *p < 0.05

Regarding known-group validity, participants with comorbidity scored worse on the PAI-CY than those without comorbidity (*p* < 0.001, respectively). Moreover, participants with severe VI or blindness scored significantly worse than participants with no VI (*p* = 0.030 and *p* = 0.023, respectively). A trend for worsening scores with increasing severity of VI was observed. No significant difference in PAI-CY scores was found for age and sex (Fig. 3).

Most items showed satisfactory test–retest reliability (Table 2), although for two items (“looking at something for a longer period of time” and “reading books together”) agreement was < 60% and for one item (“reacting to visual stimuli”) weighted kappa was 0.6. The ICC between test and retest data was 0.920 (95% confidence interval 0.880–0.946).

## Discussion

With these acceptable psychometric properties, the PAI-CY 0–2 years should be useful in low vision services, in which the perspectives from parents of very young children with VI can now systematically be assessed. After deleting 4 items, the remaining 27 items showed satisfactory model fit and a unidimensional scale measuring developmental and participation needs. Although we observed some violations in IRT assumptions, it was decided not to delete any items at this stage. Furthermore, removal of the worst performing item (“reacting to sudden actions”) caused more violations in assumptions and worsened model fit. The PAI-CY 0–2 year seems able to discriminate between participants with varying levels of clinical conditions, i.e., comorbidity and degree of VI.

Not many instruments focusing on functioning, participation, and/or quality of life are available for children this young age. Instruments for children with disabilities [26] or visual impairment are even more scarce [27, 28]. To our knowledge, the Children’s Visual Function Questionnaire (CVFQ) is currently the only instrument with a version for children aged below 3 years [29]. The CVFQ was developed to measure vision-related quality of life with domains related to competence, personality, family impact, and treatment difficulty imposed by specific eye conditions and might be complementary to the PAI-CY 0–2 years.

The limited sample size of our study was unavoidable; visual disabilities in early childhood are rare among the 17 million Dutch inhabitants. We took a conservative approach because of the small sample size (only four items were deleted), using less stringent criteria for item removal in order not to compromise face and content validity. Deleting potentially relevant and informative items prior to the availability of larger samples could be counterproductive in the long term. The planned use of the PAI-CY 0–2 years by Dutch low vision services and in research as a patient-reported outcome measure enables confirming its psychometric properties. New applications of IRT for small sample sizes, using longitudinal data, should be considered [30]. This might facilitate further item reduction, increase precision, and improve user-friendliness. Moreover, feasibility and acceptability of the questionnaire to respondents and professionals in clinical care should be monitored.
